# Management of acute cholecystitis with contained perforation with endoscopic ultrasound-guided gallbladder drainage

**DOI:** 10.1055/a-2869-7038

**Published:** 2026-06-29

**Authors:** Francesco Auriemma, Roberto Leone, Danilo Paduano, Gianluca Franchellucci, Carmine Gentile, Benedetto Mangiavillano

**Affiliations:** 1 Gastrointestinal Endoscopy UnitHumanitas Mater DominiCastellanza (VA)Italy; 2 Gastroenterology and Digestive Endoscopy Unit 9268 IRCCS Humanitas Research HospitalRozzanoMilanItaly; 3 Department of Biomedical Sciences 150525 Humanitas UniversityPieve EmanueleMilanItaly

Endoscopic ultrasound–guided gallbladder drainage (EUS-GBD) is an established alternative for treating acute cholecystitis (AC) in poor surgical candidates. 1​ Advanced age and cardiovascular comorbidities are known risk factors for gallbladder perforation; 2​ ​3​ however, the optimal management of AC with contained gallbladder perforation (CGP) remains unclear, with percutaneous drainage traditionally considered the treatment of choice because of safety concerns with EUS-GBD 3​ .

An 82-year-old women with multiple comorbidities (hypertension, atrial fibrillation, and type 2 diabetes) presented to the Emergency Department with right hypochondriac pain, nausea, and vomiting. On presentation, vital signs were normal and physical examination revealed right upper quadrant tenderness and a positive Murphy sign. Laboratory test results showed leukocytosis, elevated C-reactive protein and abnormal liver biochemistry (the total bilirubin was mildly elevated to 1.53 mg/dL, and the gamma-glutamyltransferase was elevated to 288 IU/L). An initial computed tomographic (CT) scan ( Fig. 1 ) showed a distended gallbladder containing multiple radiopaque gallstones and a 6-mm gallstone in the common bile duct with upstream dilation of the intrahepatic bile ducts.

**Fig. 1:**
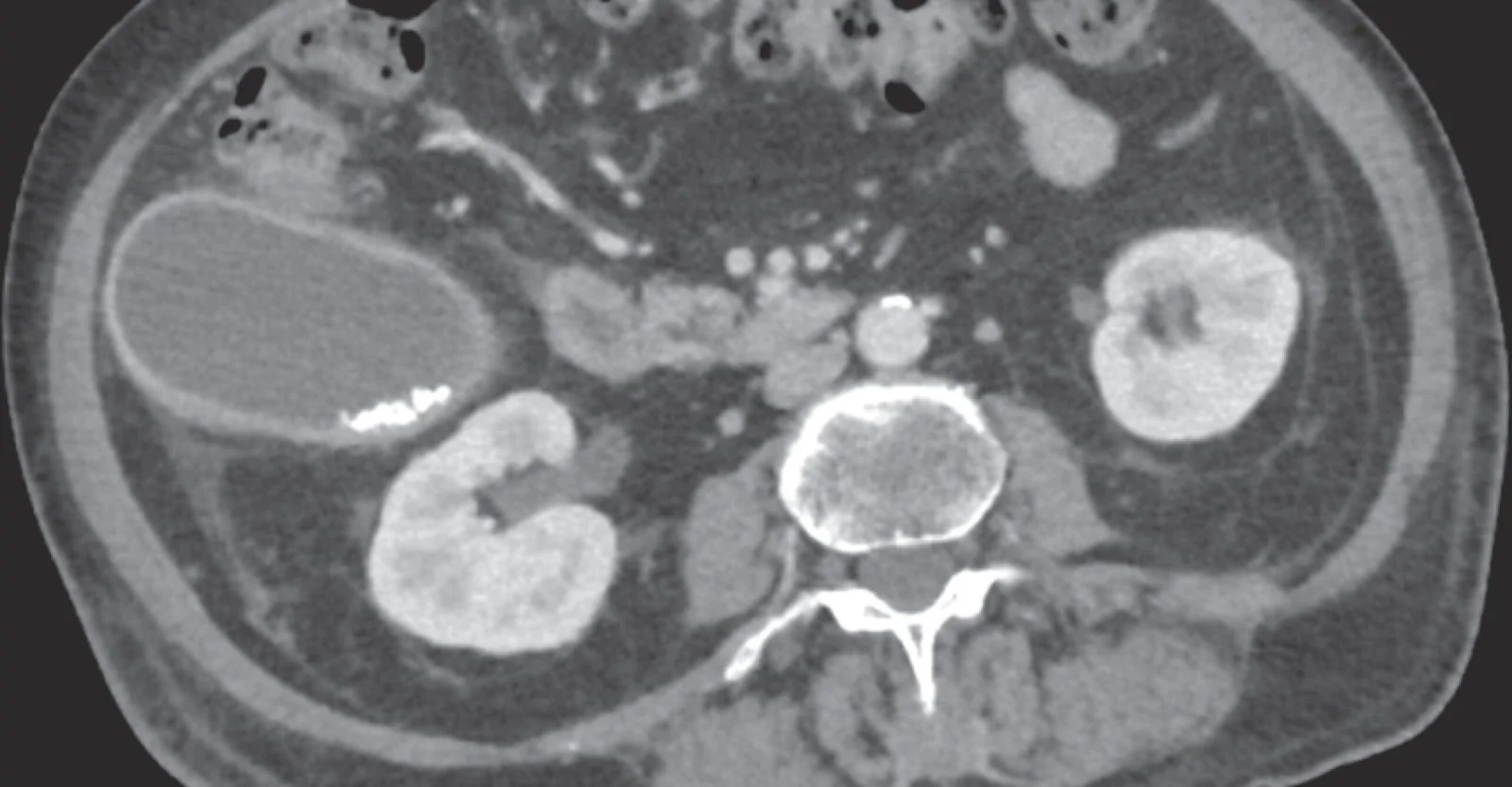
The CT scan showing dilated gallbladder with multiple gallstones.

Given the patient’s age and comorbidities, she was deemed unfit for surgery; therefore, EUS-GBD following an endoscopic retrograde cholangiopancreatography (ERCP) was chosen ( Video 1 ). During EUS examination, a focal CGP adjacent to the duodenal bulb was identified, with associated pericholecystic edema ( Fig. 2 ). After ERCP with successful removal of gallstones, EUS-guided gallbladder drainage was performed using a 10×20-mm electrocautery-enhanced lumen-apposing metal stent (LAMS; Hot-Spaxus, Taewoong, Gimpo, Korea) placed between the gallbladder and the duodenal bulb ( Fig. 3 ). A 7Fr×5-cm, plastic double pigtail stent (Advanix Biliary, Boston Scientific, Marlborough, MA) was inserted co-axially through the LAMS to reduce the risk of stent migration and occlusion. No periprocedural adverse events (AEs) occurred, and liver function tests normalized within a week. The follow-up CT scan ( Fig. 4 ) and upper GI endoscopy scheduled 20 weeks after the procedure demonstrated a patent LAMS and no late AEs.

**Video 1** Management of acute cholecystitis with contained perforation with EUS-guided gallbladder drainage.


**Fig. 2:**
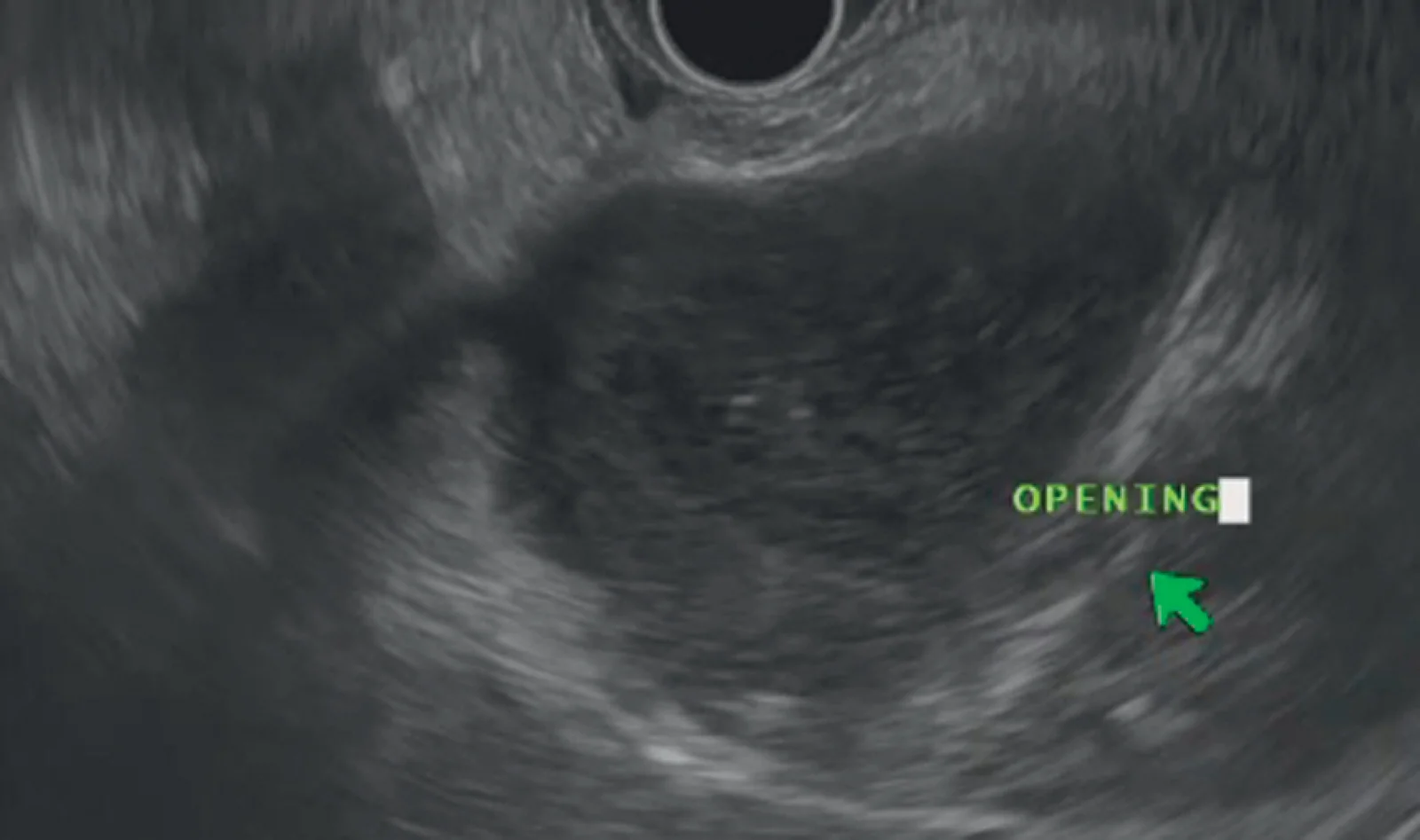
EUS image showing a contained gallbladder wall perforation.

**Fig. 3:**
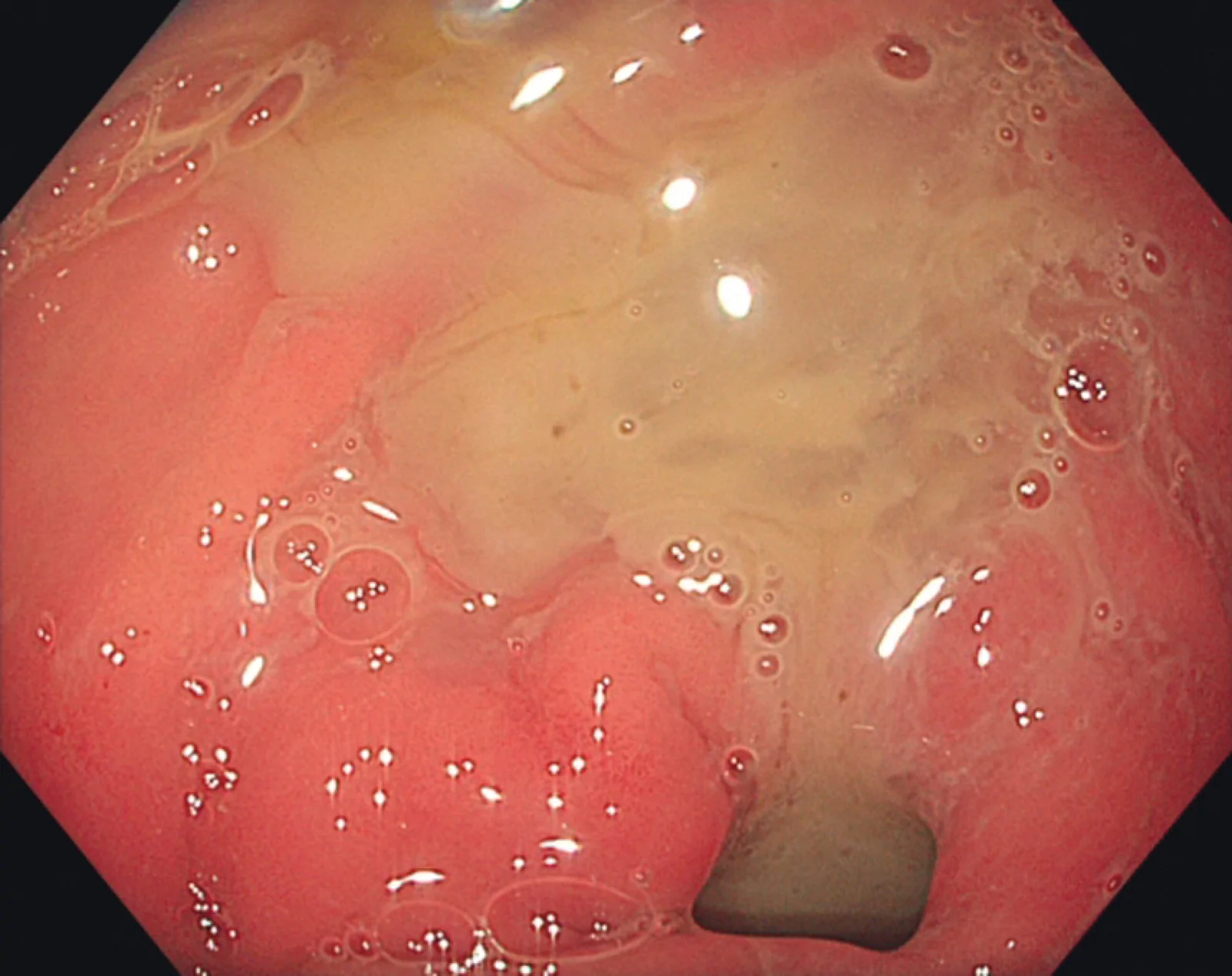
An endoscopic view of the transduodenal drainage of the gallbladder with outflow of purulent bile.

**Fig. 4:**
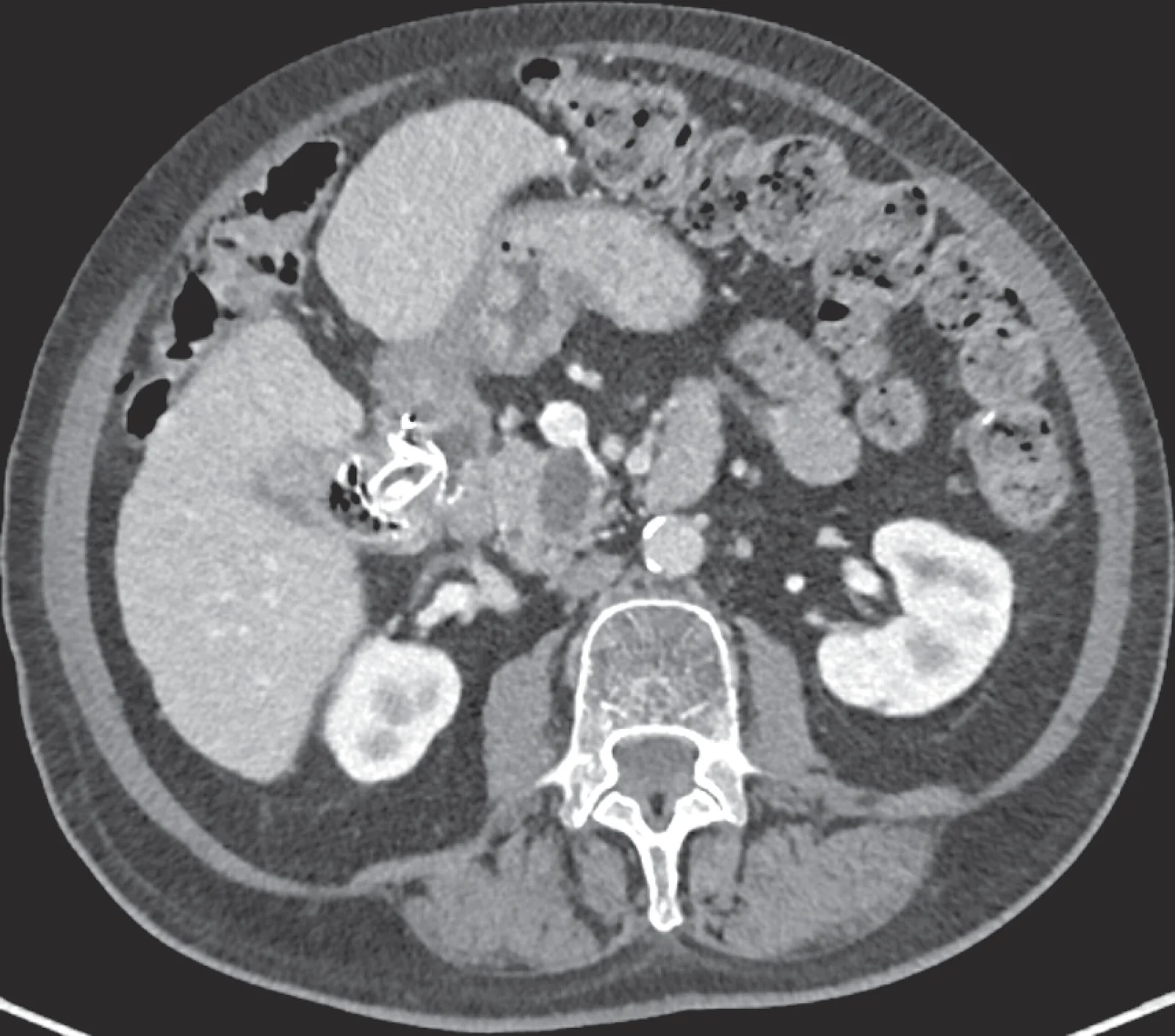
A CT scan showing the LAMS (Hot-SPAXUS, Taewoong, Gimpo, Korea) and the co-axial double pigtail plastic stent (Advanix Biliary, Boston Scientific, Marlborough, MA) in place.

EUS-guided gallbladder drainage is a safe, technically feasible and clinically effective option in selected patients with AC, even in the presence of a CGP.

Endoscopy_UCTN_Code_TTT_1AS_2AD

## Notice

This article was changed according to the erratum on August 28, 2026.

## Erratum

In the above-mentioned article the funding information was added. Correct is: The publication fee for this work was covered by the Italian Ministry of Health’s ‘Ricerca Corrente’ funding to the IRCCS Humanitas Research Hospital. This was corrected in the online version on 11. September 2026.
