# Correction: Management of acute cholecystitis with contained perforation with endoscopic ultrasound-guided gallbladder drainage

**DOI:** 10.1055/a-2944-7140

**Published:** 2026-09-11

**Authors:** Francesco Auriemma, Roberto Leone, Danilo Paduano, Gianluca Franchellucci, Carmine Gentile, Benedetto Mangiavillano

**Affiliations:** 1 Gastrointestinal Endoscopy UnitHumanitas Mater DominiCastellanza (VA)Italy; 2 Gastroenterology and Digestive Endoscopy Unit 9268 IRCCS Humanitas Research HospitalRozzanoMilanItaly; 3 Department of Biomedical Sciences 150525 Humanitas UniversityPieve EmanueleMilanItaly



In the above-mentioned article the funding information was added. Correct is: The publication fee for this work was covered by the Italian Ministry of Health’s ‘Ricerca Corrente’ funding to the IRCCS Humanitas Research Hospital. This was corrected in the online version on 28 August, 2026.

